# Addressing Data Misinterpretation in Recent Meta-Analysis

**DOI:** 10.1177/08850666251341255

**Published:** 2025-05-15

**Authors:** Kristin Alm-Kruse, Jo Kramer-Johansen

**Affiliations:** 1 Division of Prehospital services, 155272 Oslo University Hospital, Oslo, Norway; 2 Department of Anesthesia and Intensive Care, IRCCS San Raffaele Scientific Institute, Milan, Italy; 3 60504 Faculty of Medicine, Institute of Clinical Medicine, University of Oslo, Oslo, Norway

***To the Editor*,**

We are writing to address a critical error in the systematic review and meta-analysis titled “*Extracorporeal-CPR Versus Conventional-CPR for Adult Patients in Out-of-Hospital Cardiac Arrest – Systematic Review and Meta-Analysis*,”^1^ published in the Journal of Intensive Care Medicine on December 5, 2024. As authors of the one of the original research articles cited in this review, “*Outcome in Refractory Out-of-Hospital Cardiac Arrest Before and After Implementation of an ECPR Protocol*”,^2^ we are happy that our work has been found and is used in the continued collection of evidence for the best treatment for refractory out-of-hospital cardiac arrest (OHCA) patients. We have however identified a misrepresentation of our study's findings. In this call for correction, we have verified the data from our study, not the other studies included in the meta-analysis.

## Description of the Error

In the review, the data from our study were inaccurately interpreted in the following ways:

In the introduction, our results are interpreted as in favour of Extracorporeal Membrane Oxygenation (ECMO) in refractory OHCA, while our results in fact were in favour of conventional cardiopulmonary resuscitation (CCPR).

Incorrect CCPR data: In the “before” population, representing the CCPR group, survival to 30-days was 21 out of 48 patients. In the “after” population 37 out of 100 patients met the criteria for extracorporeal cardiopulmonary resuscitation (ECPR) without receiving ECMO treatment and survived to 30-days. This totals 57 out of 134 refractory OHCA receiving CCPR, surviving to 30-days.

Incorrect ECPR data: The meta-analysis incorrectly states that there were 58 ECPR 30-days survivors out of 148 cases. These 148 cases were patients meeting the criteria for potential transport with on-going CPR with intention for ECPR. Only 14 of the potential candidates received ECMO treatment. Of the ECPR treated patients, only one of the 14 patients survived 30-days or longer.

The correct use of data should thus be:

Odds for survival, ECPR: $\frac{1}{1-14}=\frac{1}{13}=0.077$

Odds for survival, CCPR: $\frac{57}{137-57}=\frac{57}{77}=0.740$

$\mathrm{The}\;\mathrm{odds}\;\mathrm{ratio}\;=\;\frac{\mathrm{Odds}\;\mathrm{for}\;\mathrm{ECPR}}{\mathrm{Odds}\;\mathrm{for}\;\mathrm{CCPR}}=\frac{0.077}{0.740}=0.104$ strongly favouring CCPR for 30 days survival in our study.

## Discussion of Possible Causes and Implications

The indication for ECPR is refractory cardiac arrest with a potentially reversible cause. We speculate that the definitions of “refractory cardiac arrest” differ between studies. In our study, we compared outcomes in the sub-groups of patients identified pre-hospitally for possible ECPR (ECPR-eligble). These sub-groups consisted of approximately 4% of the patients treated by ambulance personnel for OHCA in our setting. This is of course a highly selected group of patients and comparison of this group with all OHCA-patients will be misleading.

The incorrect usage of our data affects the conclusions drawn in the meta-analysis. Specifically, it misrepresents the effectiveness of CCPR versus ECPR in refractory OHCA. This could skew the evidence, potentially misleading future research or clinical practice. Additionally, the inflated number of ECPR-treated patients caused our data to be overemphasized, skewing the overall statistics.

We also note that our significantly worsened results on neurologic outcome at hospital discharge in the ECPR-eligible group was not included in the meta-analysis.

## Conclusion

The efficiency of ECPR relies on a complex chain of treatment, and we acknowledge that ECPR might not be a one-size fits all solution. In the Oslo-area, Norway, where we assessed the effect of ECPR, we did not find that ECPR was beneficial for the patients that met the protocol criteria. We implemented this treatment in a system where patients that met the ECPR criteria already achieved comparable good results, with 63% achieving prehospital ROSC and 44% survived to 30 days or longer. Altering this fine-tuned chain of treatment resulted in more survivors with a reduced neurological function. Our results are important because they argue the importance of assessing the effect of new treatments within each system, to make sure it is beneficial for the patients, and do not induce harm.

Maintaining the integrity of scientific literature is paramount, and it is worrisome when three independent authors have reviewed the data for the meta-analysis, all ending up producing the same errors in the data extraction process. We are willing to collaborate with the authors of the review and the journal to ensure proper modification.

## Abbreviations

CPR: Cardiopulmonary Resuscitation
CCPR: Conventional Cardiopulmonary Resuscitation
ECMO: Extracorporeal Membrane Oxygenation
ECPR: ECMO Cardiopulmonary Resuscitation

## References

[1] Reddy S Garcia S Hostetter LJ , et al. Extracorporeal-CPR versus conventional-CPR for adult patients in out of hospital cardiac arrest. Systematic review and meta-analysis. J Intensive Care Med. 2025;40(2):207-217. 39635840 10.1177/08850666241303851

[2] Alm-Kruse K Sørensen G Osbakk SA , et al. Outcome in refractory out-of-hospital cardiac arrest before and after implementation of an ECPR protocol. Resuscitation. 2021;162:35-42. 33581226 10.1016/j.resuscitation.2021.01.038

